# Low Intensive Lifestyle Modification in Young Adults With Metabolic Syndrome A Community-Based Interventional Study in Taiwan

**DOI:** 10.1097/MD.0000000000000916

**Published:** 2015-06-05

**Authors:** Yi-Lien Liu, Chia-Wen Lu, Leiyu Shi, Yiing-Mei Liou, Long-Teng Lee, Kuo-Chin Huang

**Affiliations:** From the Department of Family Medicine, National Taiwan University Hospital (Y-LL, C-WL, L-TL, K-CH); Department of Family Medicine, Min-Sheng General Hospital, Taoyuan City, Taiwan (Y-LL); Department of Health Policy and Management, Johns Hopkins School of Public Health, MD, USA (LS); Institute of Clinical and Community Health Nursing, and Laboratory of Physical Activity & Obesity Prevention, National Yang-Ming University Taipei (Y-ML); and Graduate Institute of Clinical Medical Science, China Medical University, Taichung, Taiwan (K-CH).

## Abstract

The study aims to find whether a low intensity lifestyle modification (LILM) program was effective to achieve weight reduction and improves metabolic syndrome in young adults. Our study prospectively enrolled young adults aged 30 to 45 years with metabolic syndrome in northeastern Taiwan from June 1, 2008 to December 31, 2009. The participants in the intervention group attended a LILM program for 6 months, which included 4 interactive group discussion sessions and weekly phone contact with volunteer counselors. Participants in the comparison group, however, attended only 1 noninteractive session on diet and physical activity. The main outcomes measured the weight reduction and prevalence of metabolic syndrome in intervention and comparison groups. Generalized estimating equation modeling was used to analyze the effects at baseline, during the study, and postcompletion of the program. Compared with comparison group, the intervention group showed significantly greater reductions in body weight (−2.95 ± 3.52 vs −0.76 ± 2.76 kg, *P* < 0.0001) and body mass index (−1.03 ± 1.25 vs −0.30 ± 1.16 kg/m^2^, *P* < 0.0001). After adjustment for potential confounders, a modest decrease in body weight resulted in a statistically significant 43.32% resolution in the prevalence of metabolic syndrome in the intervention group compared with 33.64% in the comparison group (*P* < 0.01).

The 6-month LILM program is not only effective in weight reduction but also an efficient intervention tool of metabolic syndrome in a community setting. The program with restricted manpower and limited medical resources can be practically transferred into primary care in rural area.

## INTRODCTION

The prevalence of obesity has dramatically increased in recent years.^1^ Obesity is associated with increased coronary heart disease, type 2 diabetes, metabolic syndrome (MetS), and all-causes mortality.^2,3^ The high prevalence of obesity raised not only public health issue but also economic burden.^4^

To be against the worldwide trend of obesity, a series of studies focused on the lifestyle modification (LM) programs and offered obese patients intensive counseling and behavioral interventions to promote sustained weight loss.^5^ However, obesity is not sufficient addressed in community settings while primary care providers infrequently offer counseling in practice levels.^6^

There were some studies elucidated from weight reduction to regression of MetS. After 6 to 24 months diet with/without exercise intervention, the reduction in the prevalence of MetS was 35% to 52.4% in intervention group.^7,8^ It must be noted that the previous studies provided a rather intensive LM to decrease the values of metabolic components, which is unlikely to be practical as a part of routine primary care in many countries.^9,10^

To investigate the compliance and adherence, it has been recognized that high attendance in an LM program positively impacts the reduction of risks related to MetS in a community setting.^11^ A number of studies report that regular follow-up is more likely to maintain LM while low compliance leads to unsuccessful improvement of MetS. Long-duration interventions tend to have attrition rates as high as 40% and for weight control programs, more than 50% dropout might be expected in community-based interventions.^12–14^ Otherwise, the development of social networks with peer support strategies using volunteer-based approaches may lift the level of compliance to LM.^15–17^

Some studies have shown that coronary heart disease may progress rapidly in young adults with MetS.^18,19^ Early identification and intervention in MetS may help prevent MetS-related disease progression, lessening the considerable economic impact of treating long-term chronic condition.^20^

The study aimed to examine whether an LM program with low intensity and volunteers-based approach can achieve weight reduction in a rural area. Also for the highly correlation between weight reduction and regression of MetS, the study targeted at young adults with MetS to elucidate the importance of early intervention.

## METHODS

### Study Settings

The study consisted of 2 phases. The specific objectives of phase I were to identify young adults aged 30 to 45 years who exhibit potential characteristics of MetS and to evaluate the lifestyle pattern of individuals with MetS. In phase II, the low intensity lifestyle modification (LILM) program, consisted of a 6-month, quasi-experimental, community level study. The subjects were young adults with MetS at 12 rural primary healthcare centers in northeastern Taiwan. Individuals who had been treated with antidiabetic, antihypertensive, or antihypertriglyceridemia agents were excluded. Individuals who were unwilling to participate in the intervention program were not enrolled. Women were ineligible if they were pregnant during the previous 6 months or breast-feeding. The participants were considered to have MetS if they met ≥3 of the following criteria: waist circumference (WC) ≥90 cm in men or ≥80 cm in women; serum triglycerides ≥1.69 mmol/L; high density lipoprotein cholesterol (HDL-C) < 1.03 mmol/L in men or <1.29 mmol/L in women; systolic blood pressure ≥130 and/or diastolic blood pressure ≥85 mm Hg; and fasting glucose ≥5.55 mmol/L.

### Allocation and Data Collection

The unmedicated MetS subjects were assigned to either an intervention group or a comparison group according to their township of residence. Each participant was given a detailed self-report questionnaire asking questions about their socio-demographic characteristics, including age, gender, educational attainment, marital status, smoking, drinking, betel nut chewing, physical activity, physician-diagnosed diseases, and medication history. This information was collected when the participants underwent a complete physical examination.

Participants’ height and weight were measured without shoes and in light clothing. WC was measured with a nonelastic tape measure, halfway between the last floating rib and the iliac crest.^21^ Blood pressure was measured twice with the automated blood pressure measurement device (Colin BP-203RV II). The average of 2 measurements taken at a 2- or 3-minutes interval after resting for at least 15 minutes.

In addition, the study applied the short version of the International Physical Activity Questionnaire^22,23^ to estimate total weekly physical activity level (METS/hour/week) during the past week and sorted to low, moderate, or high category of activity. Smoking and betel nut chewing were categorized as current, past, and never. Past smokers were defined as those who had abstained from smoking for ≥1 year at the time of the study. Drinking was categorized as current, never, occasional, and past. Current drinking was defined in those people drinking at least 1 drink daily. All the anthropometric, blood pressure, and biochemistry data were measured at baseline, during intervention (3 months), and at the end of intervention (6 months).

### Intervention

Participants in comparison group received a single 60-minute group session immediately following allocation only. No further contact with the interventionist occurred until after the data collection visits at 3 and 6 months. The subjects assigned to the intervention group were requested to attend a 24-week LILM program, which was provided 4 sessions at 2 weeks, 1 month, 2 months, and 4 months, respectively. Each session composed of 4 components including health status check, counseling, education, and exercise. In section of health status check, lasting for 15 minutes, the anthropometric indicators and blood pressure were measured. In counseling section, lasting for 15 minutes, the public health nurses checked individualized exercise and weight loss goals and calculated total calories and METS/hour per week based on the diary at each session. In education section, lasting for 30 minutes, the public health nurses reemphasized MetS and related chronic diseases, educated the food composition and benefit of physical activity, and gave behavioral therapy. In exercise section, lasting for 1 hour, participants were guided to warm up, stretching, rhythmic boxing aerobic dance, and cool down.

Three- and 6-months follow-up in intervention group consisted of monitoring the food and exercise diaries, giving 3-minutes brief feedback and beginning a group session by public health nurses. Each volunteer who was recruited and trained before intervention supervized 7 participants of the intervention group to check weight, WC and food, and exercise diary via weekly telephone follow-up.

To standardize the intervention program protocol, public health nurses were trained by research teams in both 4 workshops and in monthly educational seminars during the intervention period. To standardized volunteers, each public health nurses trained and supervized 5 volunteers before intervention and monthly feedback during intervention. The study protocol has been approved by the institutional review board of Yilan County Health Bureau, and all participants signed the written informed consent before they entered into the research.

### Statistical Analysis

To determine the homogeneity of the general characteristics between the intervention and comparison groups, the χ^2^ test, and Student's *t* test were employed. Prevalence and mean value of components of MetS were calculated by point estimates and confidence intervals (95%CI). Generalized estimating equation model with an exchangeable correlation matrix was used to assess the repeated measurement and differences of weight loss and prevalence of MetS between the intervention group and comparison group. Time points in the analyses included baseline, during intervention (3 months), at the end of intervention (6 months). Multiple logistic regression analyses were applied to estimate the odds ratio to examine the association between MetS and weight change after adjustment Statistical significance is set at *P* < 0.01. Data were analyzed using the Statistical Package for STATA version 11.

## RESULTS

**FIGURE 1:**
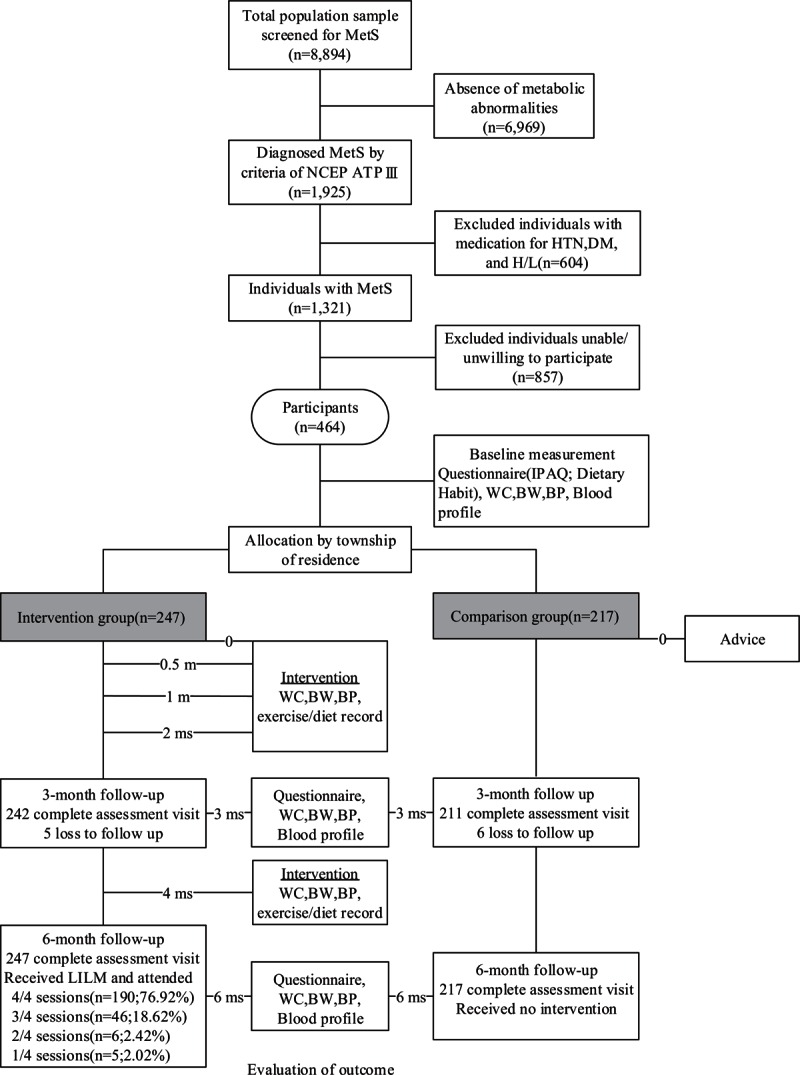
Diagram showing the flow of participants through the intervention period. BW = body weight, IPAQ = International Physical Activity Questionnaire, LILM = low intensity lifestyle modification, MetS = metabolic syndrome, WC = waist circumference.

In total, we screened 8894 subjects aged 30 to 45 years and 1925 subjects were diagnosed with MetS (21.64%) in northeastern Taiwan. Excluding 604 individuals with medication for hypertension, diabetes mellitus, or hyperlipidemia, and 857 individuals unable or unwilling to participate, the 464 eligible participants entered phase II, the LILM program. Five participants (1.21%) in the intervention group did not receive an assessment of the individual components of MetS or complete a questionnaire at 3 months in comparison to 6 participants (2.76%) in the comparison group. 190 participants (79.23%) in the intervention group undertook the group program 4 times; 46 participants (18.62%) undertook the group program 3 times; 6 participants(2.43%) undertook the group program 2 times; and 5 participants (2.02%) undertook the group program only 1 time. All the 217 participants in the comparison group did not undertake any group program (Figure 1). On average, the total manpower dedicated per participant assigned to the 6-months low intensity intervention included 2 hours of a dietitian, 4 hours of a physical activity instructor, 8 hours of an administrator, and 4 hours of a volunteer. Standard public health nurses served as dietitians. Trained volunteers served as administrator and telephone counseling providers. All interventions were performed in local public health stations or in the neighborhood of the stations. No participant reported adverse events.

The baseline characteristics and outcomes of the intervention group and the comparison group are shown in Table 1. For the baseline data, the mean age of the intervention and comparison groups were 37.32 ± 4.11 and 37.6 ± 3.81 years, respectively.

**TABLE 1:**
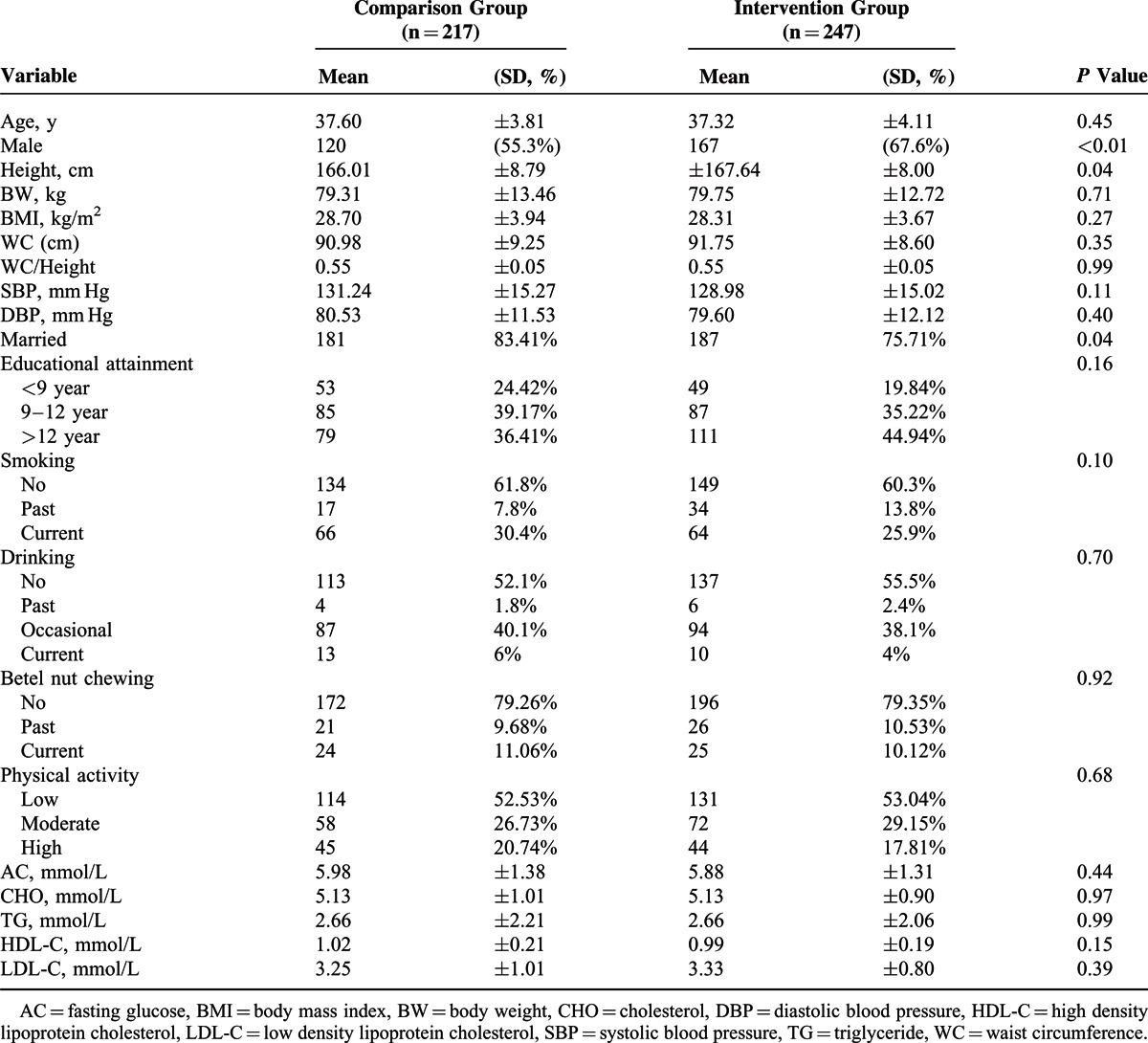
Baseline Characteristics of the Participants (N = 464)

The amount of weight loss among participants in intervention and comparison groups was shown in Table 2. Both groups lost a modest amount of weight at 3 months (−2.19 ± 2.92 vs −0.80 ± 2.53 kg) and 6 months (−2.95 ± 3.52 vs −0.76 ± 2.76 kg). The intervention group lost significantly more weight than the comparison group both at 3 and 6 months. Similarly, there was a significant difference between the changes in each group over the duration of the program for body mass index at 3 months (−0.79 ± 1.21 vs −0.29 ± 1.09 kg/m^2^) and 6 months (−1.03 ± 1.25 vs −0.30 ± 1.16 kg/m^2^). In a further analysis of study participants, 31.58% of participants lost more than 5% of their baseline body weight in the intervention group compared with 9.68% participants in the comparison group, and 19.03% of participants lost more than 7% of their baseline body weight in the intervention group compared with 5.53% of participants in the comparison group after the completion of study (*P* < 0.0001).

**TABLE 2:**
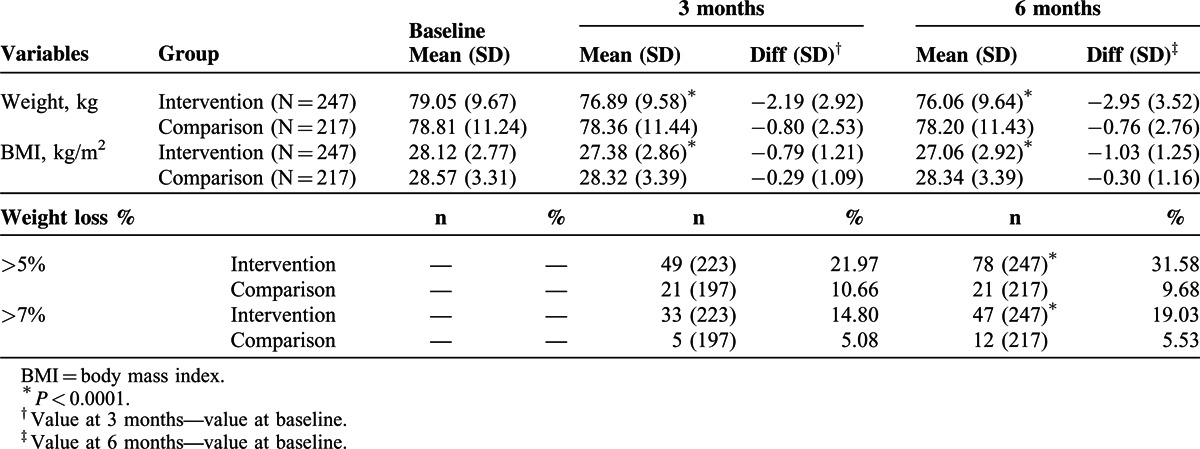
Weight Loss Among Participants Overtime Between 2 Groups (n = 464)

**FIGURE 2:**
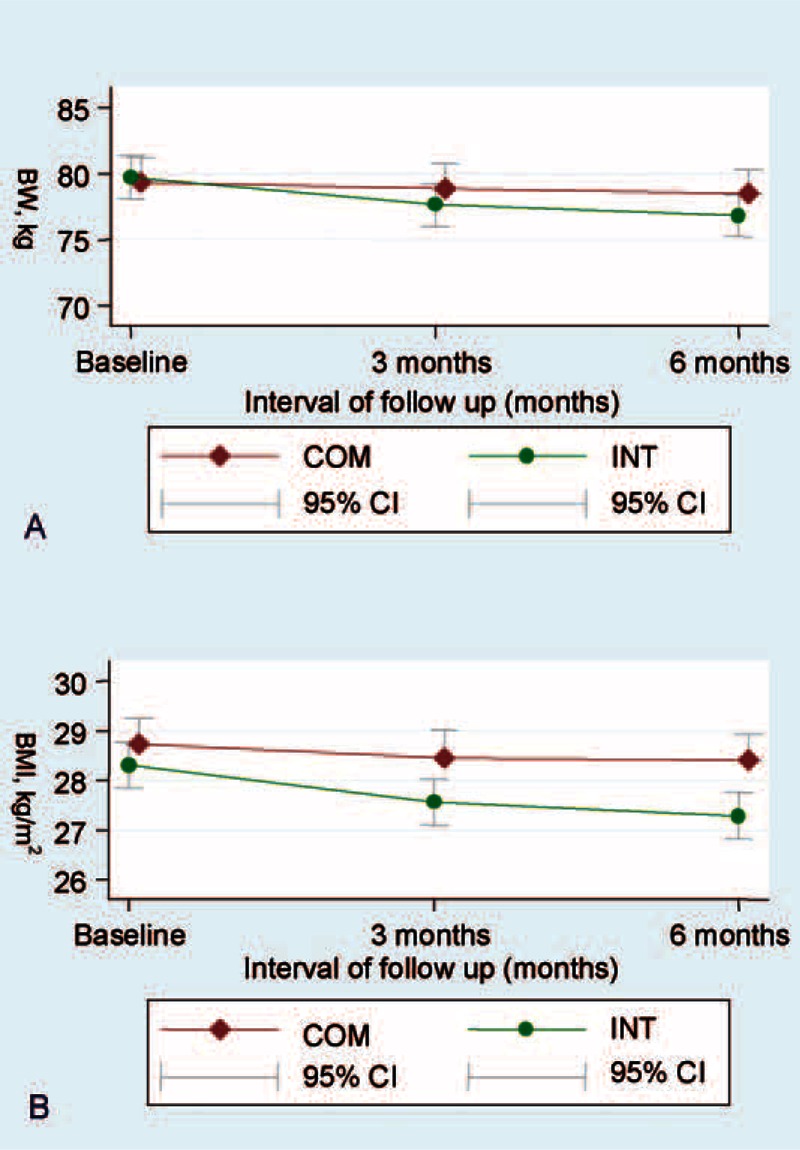
Body weight (A) and body mass index (B) in 2 groups during study period.

The trends of body weight in intervention and comparison groups were shown in Figure 2. During follow-up at 3 months, both the intervention and comparison groups showed a downward slope in body weight. After the completion of program, the intervention group kept slope downward whereas the comparison group showed a horizontal steady status.

**FIGURE 3:**
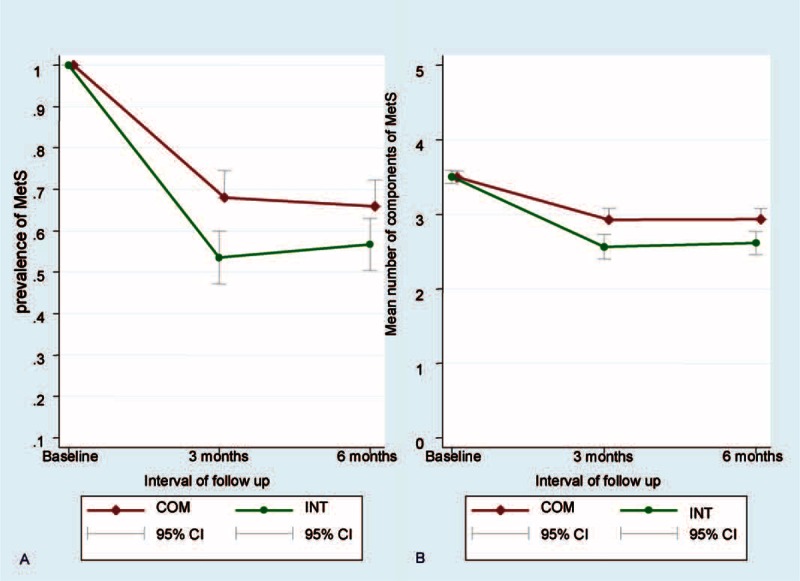
Prevalence of MetS (A) and the components of MetS (B) in 2 groups during study period.

The effect of LM on the prevalence of MetS was shown in Table 3 and Figure 3. After adjustment for age, sex, educational attainment, marital status, smoking, drinking, and betel nut chewing, a modest decrease in body weight resulted in a statistically significant 46.28% and 43.32% resolution in the prevalence of MetS in the intervention group compared with 32.23% and 33.64% in the comparison group at 3 months and 6 months, respectively (*P* < 0.01). Similarly, a modest decrease in body weight through LILM also resulted in a statistically significant 0.94 (=3.50–2.56) and 0.89 (=3.50–2.61) decreases in the mean number of components of MetS in the intervention group compared with 0.54 (=3.49–2.95) and 0.56 (=3.49–2.93) in the comparison group at 3 months and 6 months, respectively (*P* < 0.001). Additionally, results did not change after adjusting for the number of group sessions attended.

**TABLE 3:**
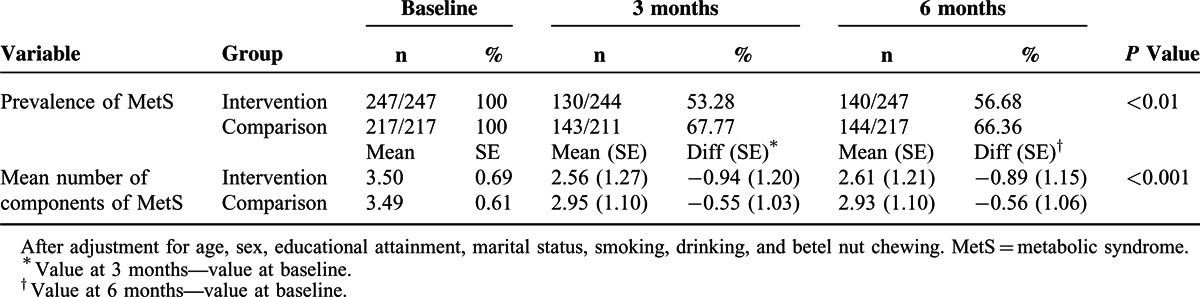
Effect of Lifestyle Modification on the Prevalence of Metabolic Syndrome

## DISCUSSION

In this study, we demonstrated that an LILM program can effectively achieve moderate weight reduction and regression of MetS in young adults at a community level. Both of the intervention and comparison groups lost a significant amount of weight at 6 months (−2.95 ± 3.52 vs −0.76 ± 2.76 kg), and the regression of MetS were 43.32% and 33.64%, respectively. Through weight reduction, the LILM program significantly decreased the prevalence and mean number of components of MetS. Importantly, the retention rate after 6 months of intervention was in excess of 95%.

Consistent with previous studies,^9,24–28^ the study elucidated the beneficial effects of LILM on body weight. With large sample size and comparison group in a rural area, the weight loss was even more modest in the intervention group than reported in previous studies^29,30^ while the intensity of intervention was lower and more practical. Comparing the slope of weight reduction between intervention and comparison group, body weight dropped in both groups initially but only kept dropping in intervention group in the latter stage. One possible explanation was that participants showed more motivation for the program in the early stage. Standard public health nurses and trained volunteers in the study prevented this enthusiasm waning in the latter half of the intervention and sustained a healthy lifestyle behavior. In previous studies, weight or WC monitoring by trained volunteers may allow individuals to help participants overcome temptations, and diet and exercise diary writing, may have increased the level of compliance to the lifestyle intervention.^15–17^

After 6 months of low intensity lifestyle modification, the prevalence of MetS declined by 43.3% in the intervention group compared with a 33.63% reduction in the comparison group (*P* < 0.0001). The intervention strategy generated an absolute risk reduction = 9.67% in the prevalence of MetS. Such an intervention seems relatively effective because it requires few patients to successfully treat 1 case with MetS. Similar studies of LM reported reductions in the prevalence of MetS, ranging from 35% to 52.4%. Esposito et al^31^ reported a 48% reduction in the prevalence of MetS after 2 years of dietary intervention with a Mediterranean-style diet when compared with the control diet. Azadbakht et al reported a 35% reduction in the prevalence of MetS after 6 months of lifestyle intervention with a DASH diet. Bihan et al^7^ reported a 52.4% reduction in the prevalence of MetS after 6 months of diet and exercise intervention. Eui Geum et al^32^ reported a 45.2% reduction in the prevalence of MetS after 6 months of diet and exercise intervention.^33^ However, these studies provided a rather intensive LM to decrease the prevalence of MetS, which is unlikely to be practical as a part of routine primary care in many countries. Similarly, diabetes prevention program by YMCA offers a low-cost approach to lifestyle diabetes prevention. Although the sample size was small and the 12 times of weekly intervention was high intensity, it provided significant weight reduction in community setting. For translation to the community at large, a less intensive intervention model involving groups of at-risk persons has been suggested as a more practical option, especially when budget and personnel constraints are factors.^34,35^

Within the comparison group, however, a reduction of 33.63% in the prevalence of MetS was also noted. One major reason could be the ecological model for behavioral changes.^36–38^ The county government ran an ongoing health promotion campaign and may have resulted in an unexpected positive outcome in the comparison group. Secondly, given the closeness of social networks in the rural community setting, the possibility of contamination followed by self-initiated lifestyle changes in the comparison group cannot be ruled out. Since more males were in the intervention group than the comparison group, we performed subgroup analyses using the generalized estimating equation models stratified by gender. In both genders, we found a greater reduction in the prevalence of MetS in the intervention group than the comparison group (*P* < 0.05). The prevalence of MetS in the intervention group were 54.5% in males and 61.3% in females; 65.0% in males and 67.0% in females in the comparison group at 6 months (data not shown).

### Strengths

First, the study enrolled a large number of participants (n = 464) from 12 rural communities. Second, the collaborative approach and partner relationship among the participants, volunteers, and trained public health nurses enabled a sustainable approach to lifestyle behavior change. Furthermore, the response rate of follow-up measurements and data collection was relatively high (>95%) at 3 and 6 months.

As residents in rural area were inconvenient access to healthcare services, counseling, and exercise facilities, they were less likely to lead a healthy lifestyle. However, the study demonstrated that a large sample size, coach-based LILM in rural area could be effective and practical achieve weight reduction with high retention rate.

### Limitations

There were some limitations in this study. First, the duration of our study was limited to 6 months, and it has been demonstrated that the efficacy of LM requires a longer follow-up time. Second, the selected 464 volunteers were probably more conscious of their own health condition than the general population. Lastly, the results were limited to a rural area in northeastern Taiwan, which cannot be directly generalized to all community setting. The 6-month LILM program for individuals with MetS in a rural community setting is effective and efficient both in weight reduction and regression of MetS. Our findings suggest that the restricted manpower, scanty medical resources and volunteers-based coaching program to initiate and sustain healthy behaviors is a practical strategy in rural area and community setting. Although short-term results are encouraging, long-term outcomes are still uncertain. In order to verify the impact and cost-effectiveness of this LILM program on lifestyle behavioral changes and clinical outcomes, a longer follow-up period is warranted.

## References

[1] Flegal KM Carroll MD Kit BK Prevalence of obesity and trends in the distribution of body mass index among US adults, 1999–2010. *JAMA* 2012; 307:491–497. 22253363 10.1001/jama.2012.39

[2] Wormser D Kaptoge S Di Angelantonio E Separate and combined associations of body-mass index and abdominal adiposity with cardiovascular disease: collaborative analysis of 58 prospective studies. *Lancet* 2011; 377:1085–1095. 21397319 10.1016/S0140-6736(11)60105-0 PMC3145074

[3] Flegal KM Kit BK Orpana H Association of all-cause mortality with overweight and obesity using standard body mass index categories: a systematic review and meta-analysis. *JAMA* 2013; 309:71–82. 23280227 10.1001/jama.2012.113905 PMC4855514

[4] Wang YC McPherson K Marsh T Health and economic burden of the projected obesity trends in the USA and the UK. *Lancet* 2011; 378:815–825. 21872750 10.1016/S0140-6736(11)60814-3

[5] Leblanc ES O’Connor E Whitlock EP Effectiveness of primary care-relevant treatments for obesity in adults: a systematic evidence review for the U.S. Preventive Services Task Force. *Ann Intern Med* 2011; 155:434–447. 21969342 10.7326/0003-4819-155-7-201110040-00006

[6] Bennett GG Warner ET Glasgow RE Obesity treatment for socioeconomically disadvantaged patients in primary care practice. *Arch Intern Med* 2012; 172:565–574. 22412073 10.1001/archinternmed.2012.1 PMC3609656

[7] Bihan H Takbou K Cohen R Impact of short-duration lifestyle intervention in collaboration with general practitioners in patients with the metabolic syndrome. *Diabetes Metab* 2009; 35:185–191. 19299181 10.1016/j.diabet.2008.11.002

[8] Oh EG Bang SY Hyun SS Effects of a 6-month lifestyle modification intervention on the cardiometabolic risk factors and health-related qualities of life in women with metabolic syndrome. *Metabolism* 2010; 59:1035–1043. 20045151 10.1016/j.metabol.2009.10.027

[9] Bo S Ciccone G Baldi C Effectiveness of a lifestyle intervention on metabolic syndrome. A randomized controlled trial. *J Gen Intern Med* 2007; 22:1695–1703. 17922167 10.1007/s11606-007-0399-6 PMC2219825

[10] Oh EG Hyun SS Kim SH A randomized controlled trial of therapeutic lifestyle modification in rural women with metabolic syndrome: a pilot study. *Metabolism* 2008; 57:255–261. 18191057 10.1016/j.metabol.2007.09.009

[11] Fujii H Haruyama Y Muto T High attendance at a lifestyle intervention program is important to reduce risks related to metabolic syndrome in middle-aged Japanese. *Tohoku J Exp Med* 2009; 219:155–164. 19776533 10.1620/tjem.219.155

[12] Keogh JB Luscombe-Marsh ND Noakes M Long-term weight maintenance and cardiovascular risk factors are not different following weight loss on carbohydrate-restricted diets high in either monounsaturated fat or protein in obese hyperinsulinaemic men and women. *Br J Nutr* 2007; 97:405–410. 17298712 10.1017/S0007114507252687

[13] van Gool CH Penninx BWJH Kempen GIJM Determinants of high and low attendance to diet and exercise interventions among overweight and obese older adults: results from the arthritis, diet, and activity promotion trial. *Contemp Clin Trials* 2006; 27:227–237. 16387555 10.1016/j.cct.2005.11.002

[14] Graffagnino CL Falko JM La Londe M Effect of a community-based weight management program on weight loss and cardiovascular disease risk factors. *Obesity* 2006; 14:280–288. 16571854 10.1038/oby.2006.36

[15] Laws R Counterweight Project T A new evidence-based model for weight management in primary care: the Counterweight Programme. *J Hum Nutr Diet* 2004; 17:191–208. 15139891 10.1111/j.1365-277X.2004.00517.x

[16] Foreyt JP Goodrick GK Factors common to successful therapy for the obese patient. *Med Sci Sports Exerc* 1991; 23:292–297. 2020266

[17] Wing RR Jeffery RW Benefits of recruiting participants with friends and increasing social support for weight loss and maintenance. *J Consult Clin Psychol* 1999; 67:132–138. 10028217 10.1037//0022-006x.67.1.132

[18] Reis JP Loria CM Lewis CE Association between duration of overall and abdominal obesity beginning in young adulthood and coronary artery calcification in middle age. *JAMA* 2013; 310:280–288. 23860986 10.1001/jama.2013.7833 PMC4226407

[19] Bal SS Khurana D Sharma A Association of metabolic syndrome with carotid atherosclerosis in the young North Indian population. *Diabetes Metab Syndr* 2011; 5:153–157. 22813569 10.1016/j.dsx.2012.02.004

[20] Kondo T Osugi S Shimokata K Metabolic syndrome and all-cause mortality, cardiac events, and cardiovascular events: a follow-up study in 25 471 young- and middle-aged Japanese men. *Eur J Cardiovasc Prev Rehabil* 2011; 18:574–580. 21450628 10.1177/1741826710389529

[21] Ness-Abramof R Apovian CM Waist circumference measurement in clinical practice. *Nutr Clin Prac* 2008; 23:397–404. 10.1177/0884533608321700 18682591

[22] Craig CL Marshall AL Sjostrom M International physical activity questionnaire: 12-country reliability and validity. *Med Sci Sports Exerc* 2003; 35:1381–1395. 12900694 10.1249/01.MSS.0000078924.61453.FB

[23] Liou YM Jwo CJ Yao KG Selection of appropriate Chinese terms to represent intensity and types of physical activity terms for use in the Taiwan version of IPAQ. *J Nurs Res* 2008; 16:252–263. 19061172 10.1097/01.jnr.0000387313.20386.0a

[24] Park TG Hong HR Lee J Lifestyle plus exercise intervention improves metabolic syndrome markers without change in adiponectin in obese girls. *Ann Nutr Metab* 2007; 51:197–203. 17587789 10.1159/000104137

[25] Villareal DT Miller BV 3rd Banks M Effect of lifestyle intervention on metabolic coronary heart disease risk factors in obese older adults. *Am J Clin Nutr* 2006; 84:1317–1323. 17158411 10.1093/ajcn/84.6.1317

[26] Orchard TJ Temprosa M Goldberg R The effect of metformin and intensive lifestyle intervention on the metabolic syndrome: the Diabetes Prevention Program randomized trial. *Ann Intern Med* 2005; 142:611–619. 15838067 10.7326/0003-4819-142-8-200504190-00009 PMC2505046

[27] Yamashiro T Nishikawa T Isami S The effect of group-based lifestyle interventions on risk factors and insulin resistance in subjects at risk for metabolic syndrome: the Tabaruzaka Study 1. *Diabetes Obes Metab* 2010; 12:790–797. 20649631 10.1111/j.1463-1326.2010.01236.x

[28] Vadheim LM Brewer KA Kassner DR Effectiveness of a lifestyle intervention program among persons at high risk for cardiovascular disease and diabetes in a rural community. *J Rural Health* 2010; 26:266–272. 20633095 10.1111/j.1748-0361.2010.00288.x

[29] Muzio F Mondazzi L Sommariva D Branchi A Long-term effects of low-calorie diet on the metabolic syndrome in obese nondiabetic patients. *Diabetes Care* 2005; 28:1485. 15920073 10.2337/diacare.28.6.1485

[30] Hofso D Nordstrand N Johnson LK Obesity-related cardiovascular risk factors after weight loss: a clinical trial comparing gastric bypass surgery and intensive lifestyle intervention. *Eur J Endocrinol* 2010; 163:735–745. 20798226 10.1530/EJE-10-0514 PMC2950661

[31] Esposito K Marfella R Ciotola M Effect of a mediterranean-style diet on endothelial dysfunction and markers of vascular inflammation in the metabolic syndrome: a randomized trial. *JAMA* 2004; 292:1440–1446. 15383514 10.1001/jama.292.12.1440

[32] Eui Geum O So Youn B Sa Saeng H Effects of a 6-month lifestyle modification intervention on the cardiometabolic risk factors and health-related qualities of life in women with metabolic syndrome. *Metabolism* 2010; 59:1035–1043. 20045151 10.1016/j.metabol.2009.10.027

[33] Azadbakht L Mirmiran P Esmaillzadeh A Beneficial effects of a dietary approaches to stop hypertension eating plan on features of the metabolic syndrome. *Diabetes Care* 2005; 28:2823–2831. 16306540 10.2337/diacare.28.12.2823

[34] Ma J Yank V Xiao L Translating the Diabetes Prevention Program lifestyle intervention for weight loss into primary care: a randomized trial. *JAMA Intern Med* 2013; 173:113–121. 23229846 10.1001/2013.jamainternmed.987 PMC3856315

[35] Ackermann RT Finch EA Brizendine E Translating the Diabetes Prevention Program into the community. The DEPLOY Pilot Study. *Am J Prev Med* 2008; 35:357–363. 18779029 10.1016/j.amepre.2008.06.035 PMC2610485

[36] Larson NI Story MT Nelson MC Neighborhood environments: disparities in access to healthy foods in the U.S. *Am J Prev Med* 2009; 36:74–81. 18977112 10.1016/j.amepre.2008.09.025

[37] Cutts BB Darby KJ Boone CG City structure, obesity, and environmental justice: an integrated analysis of physical and social barriers to walkable streets and park access. *Soc Sci Med* 2009; 69:1314–1322. 19751959 10.1016/j.socscimed.2009.08.020

[38] Caprio S Daniels SR Drewnowski A Influence of race, ethnicity, and culture on childhood obesity: implications for prevention and treatment. *Diabetes Care* 2008; 31:2211–2221. 18955718 10.2337/dc08-9024 PMC2571048

